# Atomoxetine and circadian gene expression in human dermal fibroblasts from study participants with a diagnosis of attention-deficit hyperactivity disorder

**DOI:** 10.1007/s00702-021-02373-5

**Published:** 2021-07-17

**Authors:** Frank Faltraco, Denise Palm, Adriana Uzoni, Frederick Simon, Oliver Tucha, Johannes Thome

**Affiliations:** Department of Psychiatry and Psychotherapy, University Medical Centre Rostock, Rostock, Gehlsheimer Str. 20, 18147 Rostock, Germany

**Keywords:** Atomoxetine, Human dermal fibroblasts, Circadian rhythm

## Abstract

Atomoxetine (ATO) is a second line medication for attention-deficit hyperactivity disorder (ADHD). We proposed that part of the therapeutic profile of ATO may be through circadian rhythm modulation. Thus, the aim of this study was to investigate the circadian gene expression in primary human-derived dermal fibroblast cultures (HDF) after ATO exposure. We analyzed circadian preference, behavioral circadian and sleep parameters as well as the circadian gene expression in a cohort of healthy controls and participants with a diagnosis of ADHD. Circadian preference was evaluated with German Morningness-Eveningness-Questionnaire (D-MEQ) and rhythms of sleep/wake behavior were assessed via actigraphy. After *ex vivo* exposure to different ATO concentrations in HDF cultures, the rhythmicity of circadian gene expression was analyzed via qRT-PCR. No statistical significant effect of both groups (healthy controls, ADHD group) for mid-sleep on weekend days, mid-sleep on weekdays, social jetlag, sleep WASO and total number of wake bouts was observed. D-MEQ scores indicated that healthy controls had no evening preference, whereas subjects with ADHD displayed both definitive and moderate evening preferences. ATO induced the rhythmicity of *Clock* in the ADHD group. This effect, however, was not observed in HDF cultures of healthy controls. *Bmal1* and *Per2* expression showed a significant ZT × group interaction via mixed ANOVA. Strong positive correlations for chronotype and circadian genes were observed for *Bmal1*, *Cry1* and *Per3* among the study participants. Statistical significant different *Clock*, *Bmal1* and *Per3* expressions were observed in HDFs exposed to ATO collected from ADHD participants exhibiting neutral and moderate evening preference, as well as healthy participants with morning preferences. The results of the present study illustrate that ATO impacts on circadian function, particularly on *Clock, Bmal1* and *Per2* gene expression.

## Introduction

Circadian rhythmicity is a fundamental feature of mammalian physiology that has developed under the continuous evolutionary pressure of energy conservation and efficiency. Evolution has fine-tuned the inner clock to anticipate and respond to several environmental stimuli. In modern technological societies, circadian entrainment is subjected to alterations from artificial lighting as well as lifestyle changes. These alterations lead to the increased development of disorders, e.g. attention-deficit hyperactivity disorder (ADHD), depression, obesity, as well as diabetes (Gerhart-Hines and Lazar 2015).

Attention deficit hyperactivity disorder (ADHD) is one of the most common childhood psychiatric disorders. The disease persists into adulthood in 40% of children with ADHD (Biederman et al. 2000). ADHD symptoms are characterized by inattention and/or hyperactivity-impulsivity. The disorder is associated with characteristic behavioral difficulties and impairments of day-to-day functioning.

ADHD is associated with circadian rhythm disturbances as well as disturbances in the circadian genes (*Clock, Bmal1, Per1-3, Cry1-2*). Individuals with a diagnosis of ADHD often exhibit disturbances in the circadian day-night rhythm (Rybak et al. 2007). Adults with a diagnosis of ADHD often have difficulties falling asleep as well as sleep through the night (Konofal et al. 2010; Philipsen et al. 2006).

Several genome-wide-association-studies (GWASs) have detected associations between chronotype and genotype (Gaspar^ 2017^; Hu et al. 2016; Jones^ 2016^; Kalmbach et al. 2017; Lane^ 2016^). Lane et al. (2016) reported 12 loci associated with chronotype. Some of these loci are in the region of circadian genes (Lane et al. 2016). Jones et al. (2016) reported 16 loci associated with the morningness chronotype. These genes were reported to be involved in circadian rhythm and photoreception (Jones et al. 2016). Hu et al. (2016) found 15 loci associated with morningness. Seven of these loci are near to circadian genes (Hu et al. 2016). Nine of the loci reported in the Lane et al. study and the Jones et al. study were common (Kalmbach et al. 2017).

Atomoxetine (ATO) is a second line pharmacological treatment for ADHD, besides methylphenidate, which sometimes is associated with adverse reactions and intolerance. ATO is a selective noradrenaline reuptake (NET) inhibitor. It increases norepinephrine and dopamine extracellular levels in the prefrontal cortex of rats (Bymaster et al. 2002; Swanson et al. 2006). It has been found to act as an *N*-methyl-d-aspartate receptor antagonist in rat cortical neurons (Bymaster et al. 2002; Swanson et al. 2006).

The major active metabolite, 4-hydroxyatomoxetine, has been found to have sub-micromolar affinity for opioid receptors, acting as partial agonist of the kappa-opioid receptor (Bymaster et al. 2002; Swanson et al. 2006). Orally administered ATO is rapidly absorbed. First-pass metabolism is dependent on the member of the cytochrome P450—CYP2D6 activity, resulting in an absolute bioavailability of 63% for extensive metabolizers and 94% for poor metabolizers (Bymaster et al. 2002; Swanson et al. 2006). ATO reaches maximum plasma concentration within about 1–2 h of administration with the half-life varying widely between individuals, with average range of 4.5–19 h (Bymaster et al. 2002; Swanson et al. 2006). In extensive metabolisers, ATO has a plasma half-life of 5.2 h, while in poor metabolisers, 21.6 h (Bymaster et al. 2002; Swanson et al. 2006).

A study reported that daily methylphenidate and ATO treatment impacts on circadian gene protein expression in the mouse brain (Baird et al. 2013). The protein expression in the hypothalamus of mice effects on PER2 in the nucleus suprachiasmaticus (SCN) as well as on PER1 in the paraventricular nucleus after ATO daily treatment. The same group observed that PER2 expression is effected in the caudate and putamen, while PER1 expression is effected in the ventral tegmental area (Baird et al. 2013). Other studies demonstrated that ATO reduces core symptoms of ADHD and is well tolerated (Michelson 2001; Spencer^ 2001^). In a trial, Griffiths (2018) observed that 6-week treatment with ATO improved ADHD and anxiety symptoms as well as primary cognitive outcomes of response inhibition compared to placebo, in children and adolescents with ADHD. No changes were observed for sustained attention (Griffiths et al. 2018). A recent study in healthy adults demonstrated that ATO modulates reward value (Suzuki et al. 2019). It is proposed that part of the therapeutic profile of ATO may be through circadian rhythm modulation, being able to phase shift the circadian clock in mice (O’Keeffe et al. 2012). Coogan (2019) reported that patients with ADHD using ADHD-medication, methylphenidate and ATO, have lower relative amplitudes of diurnal activity rhythms, lower sleep efficiency and more nocturnal activity than both, healthy controls, and ADHD participants without medication (Coogan et al. 2019).

In this study, we investigate the effects of ATO on circadian rhythm using human dermal fibroblasts (HDFs) derived from ADHD and healthy individuals. Patient-derived HDF cultures have the advantage of being independent to light exposure and express the majority of the same receptors and pathways as neurons, including catecholamine pathways. HDFs cultures are easily established from skin biopsies and provide an advantageous model to study circadian rhythmicity and the influence of drugs on circadian gene expression (Coogan et al. 2019). We successfully established a model based on skin fibroblasts for the investigation of the circadian rhythm, particularly, circadian gene expression. The model was applied for the comparison between healthy controls and two ADHD groups, one group with medication and the other group without medication (Coogan et al. 2019).

The current study examines circadian rhythms at the behavioral and molecular levels in healthy participants and patients with ADHD, after exposure of HDFs to ATO. The goals of the study were (1) to evaluate the circadian preference, behavioral circadian as well as sleep parameters in healthy participants and patients with a diagnosis of ADHD (2) and to examine the molecular levels of clock genes after exposure of primary human-derived dermal fibroblast cultures to ATO.

Based on the assumption of the effectiveness of ATO for the treatment of ADHD, its influence on the expression of circadian genes is hypothesized. Therefore, in this study, the influence of ATO on circadian rhythmicity is investigated *in vitro*.

## Materials and methods

### Participant selection criteria

Ethical approval for the conduct of the study, including obtaining human dermal biopsy samples, was given by the ethical review committee of Rostock University (Registration-number: A2013-159) and written consent was obtained from each study participant. The study was conducted according to the ethical guidelines of the Declaration of Helsinki.

In total, 12 volunteers without a neuropsychiatric diagnosis (healthy controls) and 12 volunteers with attention-deficit hyperactivity disorder (ADHD) participating in the study were recruited via the Department of Psychiatry and Psychotherapy, University Medical Centre Rostock. All ADHD subjects meeting DSM-IV and ICD-10 criteria diagnosed by experienced psychiatrists in advance. The healthy control group was recruited of acquaintances of people involved in the study.

Human dermal fibroblasts (HDF) were obtained from skin biopsies from dorsal forearm from ADHD patients and healthy control volunteers. Only adult individuals, able to give informed consent, were included. Healthy controls without a history of childhood and adult ADHD were matched for sex and age. Patients with more severe psychiatric symptoms were excluded, as were shift workers. Screening for ADHD symptoms was done by assessment of symptoms according to DSM-IV and ICD-10 criteria. Additionally, the following psychometric tests were used to confirm ADHD diagnosis: Wender Utah Rating Scale (WURS-k), SKIDI and II (Structured clinical interview), DIVA 2.0 (Structured diagnostic interview), CAARS (Conners’ Adult ADHD Rating Scales) and PSQI (Pittsburgh Sleep Quality Index). The IQ of the healthy control group and volunteers with ADHD diagnosis was measured using the Multiple Choice Word Test (MWT). The chronotype of the participants was determined by the Morning-Eveningness-Questionnaire, German Version (D-MEQ). No special cognitive testing was implemented in the study.

Comorbidities were observed: 16.7% of participants with ADHD diagnosis has additionally adipositas, 8.3% has additionally addiction disorder, and 25% has additionally affective disorder. The remaining participants with ADHD diagnosis have no comorbidities.

The four manuscripts of this special issue dealing with circadian rhythmicity describe unique research questions (Faltraco et al. 2021a, b; Palm et al. 2021). Although some samples have been used for more than one research question, the overall sample composition differs from each other and thus is different for each study. Experiments differ substantially in their conditions, thus, they each investigate unique cellular biochemical pathways.

### Actigraphy

To obtain objective measures of participants’ sleep and circadian rhythm function, the rest–activity pattern of participants was recorded using wrist-worn actigraphs (Actiwatch 2, Philips Respironics, USA). Actigraphs were worn on the non-dominant wrist for a period of at least 7 consecutive days. The recording interval of the device was set at 60-s epochs. Data occurring before the first and after the final midnight of each record were excluded, ensuring at least 6 complete days for each participant, with a complete weekend included in each record.

### Tissue isolation and fibroblast cell culture

Human dermal fibroblasts (HDF) were isolated and cultured as described previously (Takashima 1998). Fibroblasts were cultivated (37 °C, 5% CO_2_) in Dulbecco’s Modified Eagle Medium DMEM (Gibco, Thermo Fisher, UK)/1 mg/ml Liberase TM (Roche, Germany) containing 100 units/ml penicillin, 100 µg/ml streptomycin (Gibco, Thermo Fisher, UK) and 10% fetal bovine serum FBS (Gibco, Thermo Fisher, UK).

### Measurement of cell viability

Upon confluency of the respective primary fibroblast cell culture from each participant, cells were incubated with 0 µM, 0.2 µM and 0.58 µM ATO (Atomoxetine hydrochloride, European Pharmacopoeia (EP) Reference Standard, Merck, Germany) in DMEM with 5% FBS. Following 24 h, cell viability was measured using the Trypan Blue Exclusion Test (Strober 2015).

### Measurement of circadian gene expression

Upon confluency of the respective primary fibroblast cell culture from each participant, eight culture flask replicates were prepared and cells were incubated with either 0.2 µM or 0.58 µM ATO (Atomoxetine hydrochloride, European Pharmacopoeia (EP) Reference Standard, Merck, Germany) in DMEM with 5% FBS for 24 h. Cultures without ATO were used as a negative control. The following day, the cells were synchronized with 100 nM dexamethasone (Sigma-Aldrich, Germany) for 30 min. Samples were harvested every fourth hour after synchronization for a period of 28 h in GTC lysis buffer (4.5 M guanidinium thiocyanate, 0,5% sodium-*N*-lauryl sarcosine, 25 mM tri-sodium citrate, 0.1 M betamercaptoethanol) and stored at − 80 °C. mRNA was isolated and purified with RNeasy Plus Mini Kit (Qiagen, Germany) as well as subjected to reverse transcription using the Superscript III First-Strand Synthesis System (Invitrogen, Germany). Gene expression of *Clock, Bmal1, Cry1, Per1, Per2,* and *Per3* as well as housekeeping genes, *Rpl13A, Rpl19A, GAPDH*, was measured by real-time quantitative reverse transcriptase polymerase chain reaction (qRT-PCR) with CFX Connect™ Real-Time PCR Detection System (Biorad, Germany). All primers were purchased from Eurofins (Alameda, CA). The oligonucleotide sequences are presented in Table 1. The qRT-PCR was performed in 96-well 0.1-ml thin-wall PCR plates (Applied Biosystems) in the CFX Connect™ Real-Time PCR Detection System (Biorad, München, Germany). Each 20 µl reaction contained 10 µl PerfeCTa SYBR Green Master Mix (Quantabio, Munich, Germany), 200 nM gene-specific forward and reverse primer mix (Eurofins, Alameda, CA) and 20 ng template DNA. For normalization of the expression levels of genes of interest, the geometrical mean of expression level of housekeeping genes *Rpl13A, Rpl19A, GAPDH* from the same sample was used (Mane et al. 2008). The primer efficiency (between 1.93 and 2.00) was evaluated using the RegLinePCR v 11.0 (Heart Failure Research Center). Data were analyzed using the ΔΔCt method (Livak and Schmittgen 2001). The values were normalized to corresponding individual averages.

**Table 1:** Oligonucleotides for qRT-PCR to measure circadian gene expression

Gene	Forward primer (5′–3′)	Reverse primer (5′–3′)
*Clock*	CCAGCAGTTTCATGAGATGC	GAGGTCATTTCATAGCTGAGC
*Bmal1*	AAGGATGGCTGTTCAGCACATGA	CAAAAATCCATCTGCTGCCCTG
*Per1*	TGGGGACAACAGAACAGAGAA	AGGACACTCCTGCGACCA
*Per2*	GTATCCATTCATGCTGGGCT	TCGTTTGAACTGCGGTGAC
*Per3*	TCAGTGTTTGGTGGAAGGAA	TCTGGGTCAGCAGCTCTACA
*Cry1*	CACGAATCACAAACAGACGG	TACATCCTGGACCCCTGGT
*RPL13a*	GCCAGAAATGTTGATGCCTT	AGATGGCGGAGGTGCAG
*RPL19a*	GTGGCAAGAAGAAGGTCTGG	GCCCATCTTTGATGAGCTTC
*GAPDH*	GAAGGTGAAGGTCGGAGT	GAAGATGGTGATGGGATTTC

### Statistical methods

To determine the best-fitting linear harmonic regression, circadian gene expression data were tested for significant circadian rhythmicity, using CircWave v.1.4 software (generated by Dr. Roelof Hut; www.euclock.org). The data were set to an assumed period of 24-h and with α set at 0.05. The center-of-gravity of each best-fitting waveform in CircWave was used as the circadian acrophase, and the associated estimation error was used as the SD. Inferential statistics were carried out in SPSS (IBM Corporation). Actigraphic data were analyzed via MANCOVAs, with age, sex and in some cases, ADHD symptom severity included in the model as covariates.

qRT-PCR clock gene data were analyzed via ANOVA and mixed between-within ANOVAs. One-way ANOVA was used to assess differences of clock gene expression levels among chronotype groups. Associations between clock gene expression and chronotype obtained from healthy controls and ADHD participants were studied by Spearman’s rank order correlation. For all inferential tests, *P* < 0.05 was used to indicate a statistically significant groupwise difference. Sample sizes were calculated via GPower 3.1 software; for correlations the assumptions used were significance level of α = 0.05 and the power of 0.8 for 2 groups (ADHD, HC) with 3 measures (0 µM, 0.2 µM and 0.58 µM ATO). Although research in this field is generally scarce, we assumed that the influence of ATO on the circadian gene expression will have an effect size *d*’ = 0.5, returning a required total sample size of 21. Taking into consideration, an expected drop-out rate, *n* = 12 participants were allocated to each group.

Data were analyzed via time series statistics adequately powered by 12 samples each, which in this statistical model is mathematically sufficient and thus representative (Menet et al. 2012; Thaben and Westermark 2016).

## Results

### Demographic data

Human dermal fibroblasts (HDFs) were obtained via skin biopsy from healthy controls (HC) (4 men, 8 women; 41.50 ± 14.04 years, mean ± SD; BMI: 25.87 ± 5.42 kg/m^2^, mean ± SD) and volunteers with attention-deficit hyperactivity disorder (ADHD) (7 men, 5 women; 42.00 ± 12.88 years, mean ± SD; BMI: 25.86 ± 3.83 kg/m^2^, mean ± SD). All participants completed the Multiple-Choice Word Test (IQ score: HC: 110.25 ± 9.32, mean ± SD; ADHD participants: 109.42 ± 11.66, mean ± SD, n.s), Morningness-Eveningness-Questionnaire, German Version (D-MEQ Score: HC: 58.83 ± 8.97, mean ± SD; ADHD participants:: 48.50 ± 14.92, mean ± SD, n.s.) and Wender Utah Rating Scale, German Short Version (WURS-k Score: HC: 7.17 ± 8.19, mean ± SD; ADHD participants: 37.08 ± 16.47, mean ± SD, *p* < 0.001). The demographic data are presented in Table 2.

**Table 2:** Demographic data

Demographic data	Healthy controls *n*=12	ADHD *n*=12
Age	41.50 ± 14.04 years	42.00 ± 12.88 years
Female	8 (66.7%)	5 (35.4 %)
BMI	25.87 ± 5.42	25.86 ± 3.83
IQ-Score	110.25 ± 9.32	109.42 ± 11.66
D-MEQ	58.83 ± 8.97	58.83 ± 8.97
Chronotype	7 (58.3%) Neutral type 3 (25.0%) Moderate morning type 2 (16.7%) Definitive morning type	3 (25.0%) Neutral type 5 (41.7%) Moderate morning type 2 (16.7%) Moderate evening type 2 (16.7%) Definitive evening type
WURS-k-Score	7.17 ± 8.19***	37.08 ± 16.47***

***p< 0.001

There were no significant differences in age, BMI, IQ, D-MEQ or gender across the two study groups. D-MEQ scores indicated that HC had no evening preference, whereas ADHD patients displayed both definitive and moderate evening preferences.

### Actigraphy

Measures from the non-parametric circadian rhythm analysis were analyzed between both groups, healthy controls and ADHD participants, in a MANCOVA. Age and sex were chosen as co-variates. No statistically significant effect of group was observed (Pillai’s trace = 0.205; *F* = 0.643; *p* = 0.695; partial ETA squared = 0.205). Bonferroni post hoc correction demonstrated no significant difference for mid-sleep on weekend days (*p* = 0.774), mid-sleep on weekdays (*p* = 0.382), social jetlag (*p* = 0.553), sleep efficiency (*p* = 0.975), WASO (wakening after sleep onset; *p* = 0.927) and total number of wake bouts (*p* = 0.659).

Measures from the non-parametric circadian rhythm analysis were analyzed across the two groups, healthy controls and ADHD participants, in a MANCOVA with chronotype as co-variate. No statistically significant effect of group was observed (Pillai’s trace = 0.116; *F* = 0.351; *p* = 0.899; partial ETA squared = 0.116). A significant difference for mid-sleep on weekend days (*p* = 0.008), mid-sleep on weekdays (*p* = 0.001), but not for social jetlag (*p* = 0.928), sleep efficiency (*p* = 0.715), WASO (wakening after sleep onset; *p* = 0.925) and total number of wake bouts (*p* = 0.570) was observed. The measurements for two ADHD volunteers were not completed.

### Cell viability

The viability of the cultivated human dermal fibroblasts (HDF) after ATO incubation was compared with HDFs without ATO. The viability of cells treated with ATO (0.20 µM ATO: 96.02 ± 1.23, mean ± SD; 0.58 µM ATO: 97.03 ± 0.24, mean ± SD) was marginally decreased compared to control cells without ATO (0 µM ATO: 97.28 ± 0.32, mean ± SD).

### Circadian gene expression in human dermal fibroblasts

The expression profiles of circadian genes after incubation with 0.20 µM and 0.58 µM ATO concentrations were examined in primary fibroblasts cultured from skin biopsies collected from ADHD and healthy participants and synchronized with dexamethasone. Cultures without ATO were used as a negative control.

*Circwave* analysis revealed a significant rhythmicity of *Clock* gene (*p* = 0.019, *CircWave*) in the cultures collected from the ADHD participants incubated with 0.58 µM ATO. This effect was not observed in other HDF cultures.

Mixed between-within ANOVA analysis of circadian gene data with group as between-subject factor and time as within-subject factor showed significant main effects for time for all circadian genes (*p* < 0.01). *Bmal1* expression showed a significant zeitgeber time ZT × group interaction via mixed ANOVA (Greenhouse–Geisser corrected *F*_0,76. 21,68_ = 1.716, *p* = 0.027, partial ETA squared = 0.125), as well as *Per2* expression (Greenhouse–Geisser corrected *F*_1,32. 25,29_ = 2.308, *p* = 0.001, partial ETA squared = 0.168). No ZT × group interaction via mixed ANOVA was observed for *Clock* (Greenhouse–Geisser corrected *F*_0.41,26.67_ = 1.256, *p* = 0.183, partial ETA squared = 0.095), *Cry1* (Greenhouse–Geisser corrected *F*_0.60,20.30_ = 1.335, *p* = 0.157, partial ETA squared = 0.103), *Per1* (Greenhouse–Geisser corrected *F*_1.37,21.52_ = 1.368, *p* = 0.131, partial ETA squared = 0.098) and *Per3* (Greenhouse–Geisser corrected *F*_2.16,15.76_ = 1.554, *p* = 0.087, partial ETA squared = 0.118) expression.

**Fig. 1:**
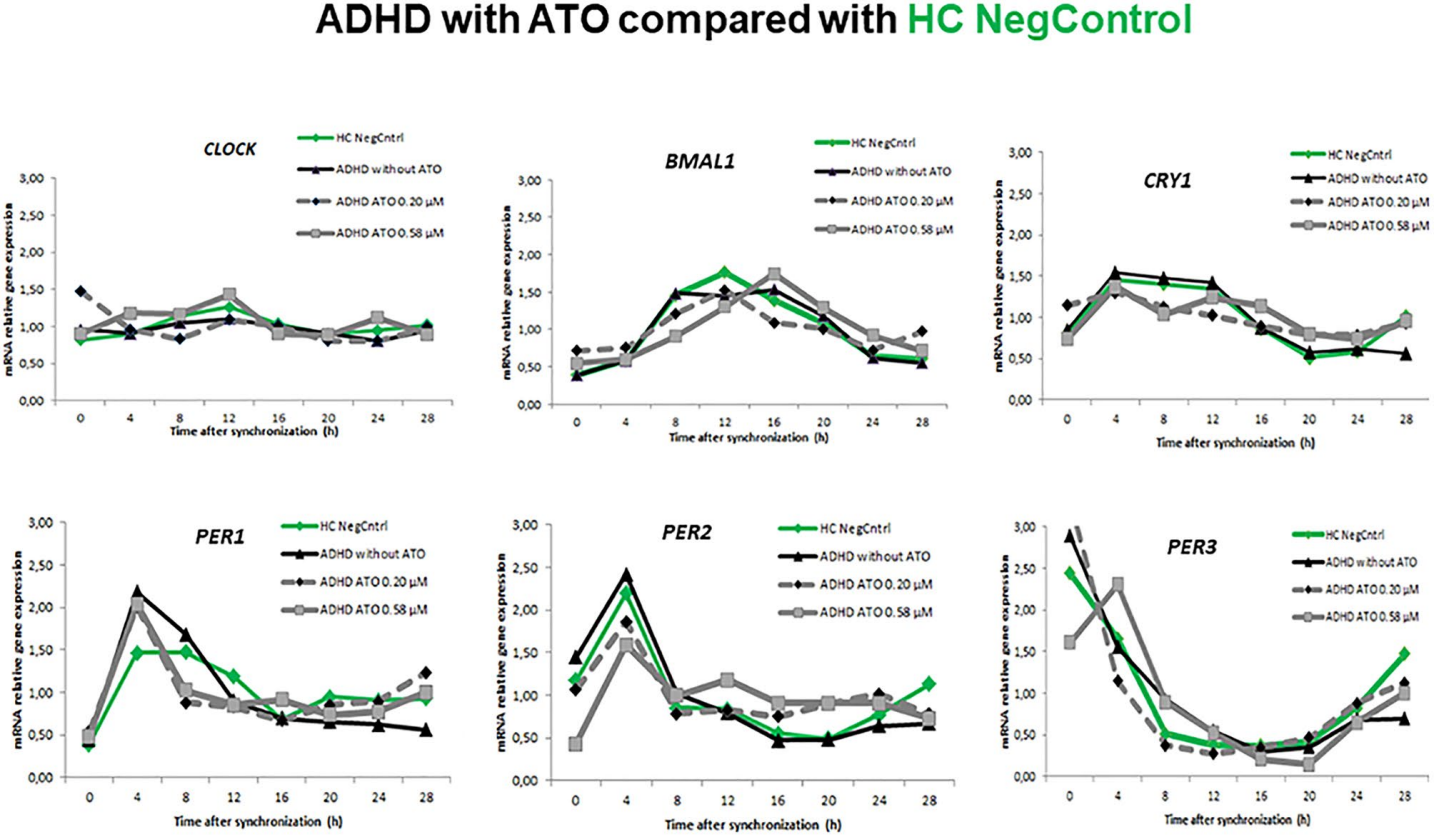
Relative mRNA gene expression of circadian genes in healthy controls (0 μM) and ADHD volunteers (0, 0.2, 0.58 μM ATO)

**Fig. 2:**
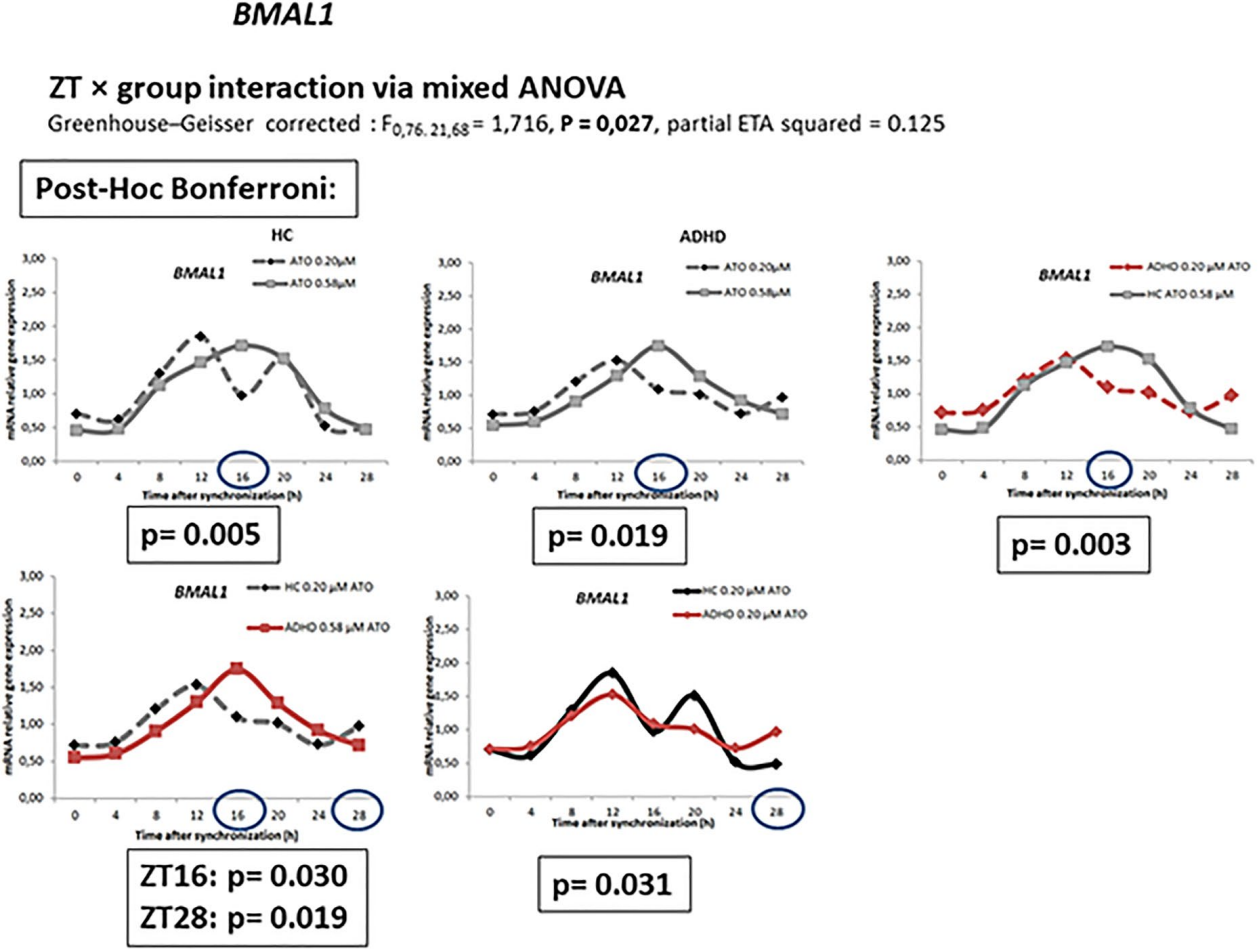
Relative mRNA gene expression of *Bmal1* in healthy controls and ADHD volunteers (0, 0.2, 0.58 μM ATO)

**Fig. 3:**
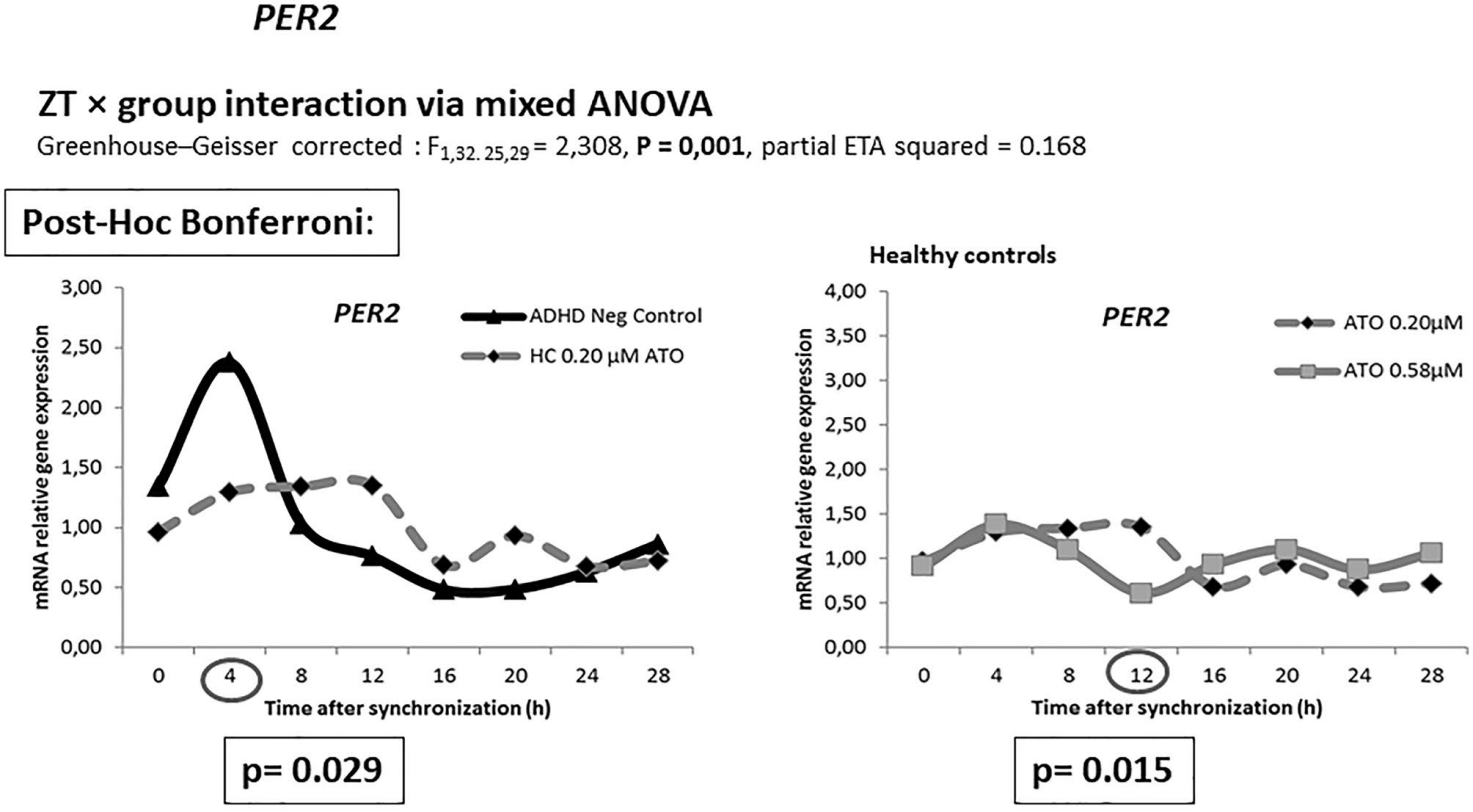
Relative gene expression of *Per2* in healthy controls and ADHD volunteers (0, 0.2, 0.58 μM ATO)

Bonferroni post hoc correction showed *Bmal1* expression at zeitgeber time ZT16 to be significant higher in cultures incubated with 0.58 µM ATO compared to those with 0.2 µM ATO among healthy controls (*p* = 0.005) and ADHD (*p* = 0.019). The same effects were observed in HDFs from volunteers with a diagnosis of ADHD with 0.58 µM (*p* = 0.003) compared to HDFs from HCs with 0.2 µM ATO. HDFs from volunteers with a diagnosis of ADHD incubated with 0.2 µM ATO resulted in different *Bmal1* expressions at ZT16 (*p* = 0.030) and ZT28 (*p* = 0.019) compared to HC cultures incubated with 0.58 µM ATO. 0.20 µM ATO induced different *Bmal1* expression between controls and ADHD group at ZT28 (*p* = 0.031). The expression of *Per2* at ZT 12 (*p* = 0.015) was significant higher in HDF cultures incubated with 0.20 µM ATO compared to those exposed to 0.58 µM ATO. The expression was lower at ZT4 (*p* = 0.029) for HDF cultures from HCs with 0.20 μM ATO compared to ADHD participants without ATO (Figs. 1, 2, 3).

**Fig. 4:**
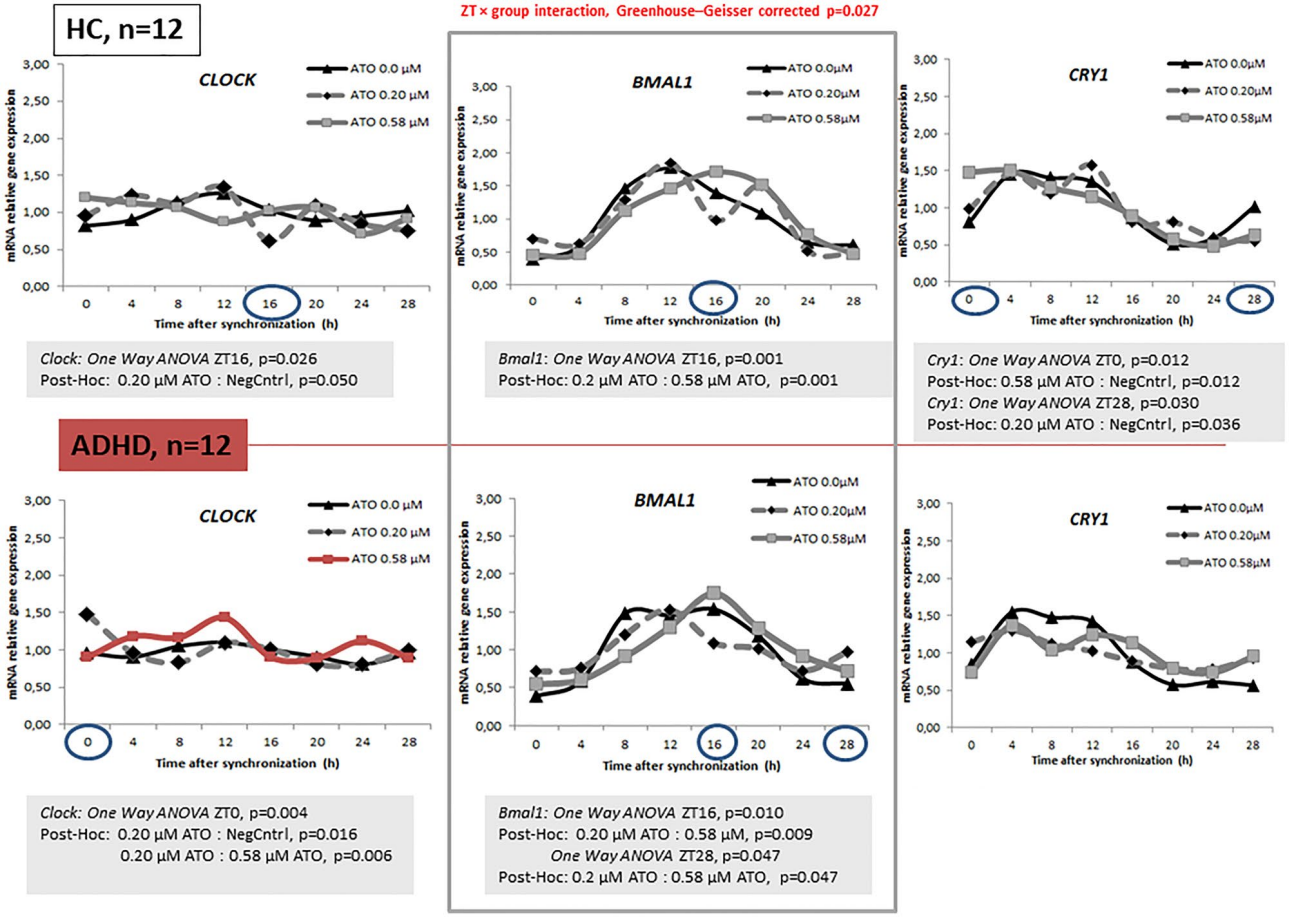
Relative mRNA gene expression of circadian genes (*Clock*, *Bmal1*, *Cry1*) in healthy controls and ADHD volunteers (0, 0.2, 0.58 μM ATO)

**Fig. 5:**
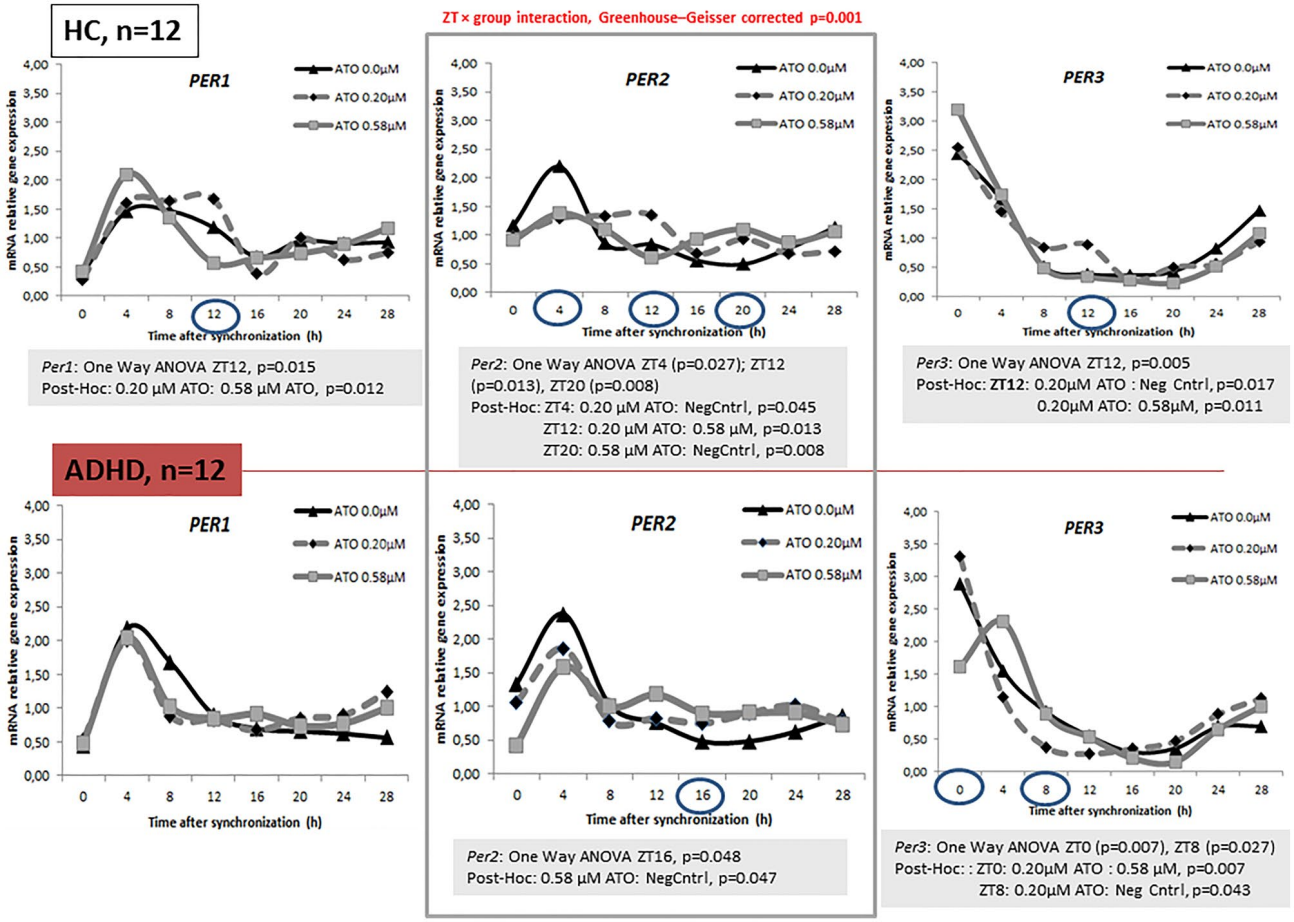
Relative mRNA gene expression of circadian genes (*Per1-3*) in healthy controls and ADHD volunteers (0, 0.2, 0.58 μM ATO)

Gene expression in healthy participants revealed a statistical significant difference between cultures incubated with ATO and negative controls (without ATO incubation), as determined by one-way ANOVA for *Clock* at ZT16 (*F* = 4.108, *p* = 0.026), *Cry1* at ZT0 (*F* = 5.069, *p* = 0.012) and ZT28 (*F* = 4.006, *p* = 0.030), *Per2* at ZT4 (*F* = 4.023, *p* = 0.027) and *Per3* at ZT 12 (*F* = 6.151, *p* = 0.005). A Bonferroni post hoc analysis revealed a significant lower expression in cultures incubated with 0.2 µM ATO compared to negative controls for *Clock* (ZT16, *p* = 0.050) and *Per2* (ZT4, *p* = 0.045). The same effect was observed for the cultures incubated with 0.58 µM ATO for *Cry1* (ZT28, *p* = 0.036). In contrast to this, the expression levels of *Cry1* at ZT0 (p = 0.012), *Per2* at ZT20 (*p* = 0.008) and *Per3* at ZT12 (*p* = 0.017) were higher in HDFs incubated with ATO compared to the cultures without ATO. The *Bmal1* (*F* = 8.086, *p* = 0.001), *Per1* (*F* = 4.773, *p* = 0.015) and *Per3* (*F* = 6.151, *p* = 0.005) expression differed between the two ATO concentrations in HDFs from HC. This was observed particularly at ZT16 for *Bmal1* (*p* = 0.001) and ZT12 for *period* genes (*Per1*, *p* = 0.012; *Per3*, *p* = 0.011) (Figs. 4, 5).

One-way ANOVA in the ADHD group revealed statistical significant differences between cultures incubated with ATO and negative controls for *Clock* (*F* = 6.703, *p* = 0.004), *Bmal1* (ZT16, *F* = 5.372, *p* = 0.010; ZT28, *F* = 3.372, *p* = 0.047), *Per2* (*F* = 3.335, *p* = 0.048) and *Per3* (ZT0, *F* = 5.836, *p* = 0.007; ZT8, *F* = 2.465, *p* = 0.027). A Bonferroni posthoc correction revealed a significant higher expression in cultures incubated with 0.2 µM ATO compared to negative controls and those with 0.58 µM, particularly at ZT0 for *Clock* (*p* = 0.016) and *Per3* (*p* = 0.007) and ZT28 for *Bmal1* (*p* = 0.047). The expression was significant lower at ZT8 for *Per3* (*p* = 0.043) and ZT16 for *Bmal1* (*p* = 0.009) and *Per2* (*p* = 0.047) in cultures incubated with 0.2 µM ATO compared to either negative controls or those with 0.58 µM ATO (Figs. 4, 5).

### Chronotype and circadian gene expression

58.3% of healthy participants displayed neutral preference, whereas 25.0% had moderate morning and 16.7% definite morning preference. 41.7% of ADHD participants displayed moderate morning and 25.0% neutral preference. Interestingly, in the ADHD group, the evening preference was represented by 16.7% definite evening and 16.7% moderate evening chronotype. There were no participants with definitive morning preference in the ADHD group.

The relationship between chronotype and circadian gene relative expressions was determined using Spearman’s rank order correlation. Strong positive correlations for chronotype and circadian genes were observed for *Bmal1, Per3* and *Cry1* among the study participants. In ADHD group chronotype and *Bmal1* gene were positively correlated in the first 8 h after dexamethasone synchronization (ZT4, *r*_*s*_ = 0.649, *p* = 0.022; ZT8, *r*_*s*_ = 0.847, *p* = 0.001). In ADHD cultures incubated with 0.58 µM ATO, a positive correlation between chronotype and *Per3* at ZT0 *(r*_*s*_ = 0.590, *p* = 0.043) was observed. Additionally, in healthy participants group was a strong positive correlation for chronotype and clock genes, particularly at ZT0 for *Per3* gene (*r*_*s*_ = 0.631, *p* = 0.028), and ZT16 for *Bmal1* (*r*_*s*_ = 0.611, *p* = 0.035) as well as *Cry1* (*r*_*s*_ = 0.591, *p* = 0.043), respectively. In HC cultures incubated with 0.58 µM ATO, the strong positive correlation for chronotype with *Bmal1* was shifted to ZT20 (*r*_*s*_ = 0.650, *p* = 0.022).

Differences of clock gene expression levels among chronotype were assessed using one-way ANOVA. Gene expression in HDFs from healthy participants revealed a statistical significant difference between chronotypes for *Per3* immediately after synchronization at ZT0 (*F* = 6.804, *p* = 0.016) and 16 h after synchronization for *Bmal1* (*F* = 4.884, *p* = 0.037). A Bonferroni post hoc correction revealed a significant lower expression of *Per3* (*p* = 0.018) and *Bmal1* (*p* = 0.040) in healthy participants exhibiting neutral chronotype compared to those with moderate morning type. One-way ANOVA in the ADHD group revealed no statistical significant clock genes differences between chronotypes.

Exposure of HDFs cultures with ATO revealed differences between chronotypes. In HC cultures incubated with 0.58 µM ATO, one-way ANOVA revealed a statistical significant gene expression differences between chronotypes for *Bmal1* (ZT0, *F* = 4.822, *p* = 0.038; ZT20, *F* = 9.168, *p* = 0.007), *Clock* (*F* = 4.491, *p* = 0.044) and *Per2* (*F* = 7.039, *p* = 0.014). Comparing healthy participants with definitive morning to those with moderate morning preference, Bonferroni post hoc correction showed significant lower *Bmal1* expression levels at ZT8 (*p* = 0.038) and ZT20 (*p* = 0.044). The expression of *Clock* at ZT24 (*p* = 0.047) was significant higher among the healthy participants with moderate morning compared to those exhibiting definitive morning preference. When compared to neutral type the expression of *Bmal1* at ZT20 (*p* = 0.006) and *Per2* at ZT28 (*p* = 0.014) was significant different in healthy participants with definitive morning, and moderate morning chronotype, respectively. In ADHD cultures incubated with 0.20 µM ATO, one-way ANOVA revealed a statistical significant differences in gene expression between chronotypes for *Per1* (*F* = 8.090, *p* = 0.008) and *Per2* (*F* = 5.278, *p* = 0.027). Bonferroni post hoc correction showed that the participants with definite evening preference had a higher *Per1* at ZT8 compared to ADHD participants with neutral (*p* = 0.013) and moderate morning preference (*p* = 0.015). Similarly, different *Per2* expressions levels were observed in ADHD participants exhibiting evening preferences at ZT4 (*p* = 0.025). Additionally, in participants with moderate evening preference, the expression of *Per2* at ZT20 was significant higher compared to those exhibiting definitive evening (*p* = 0.012), neutral (*p* = 0.038) and moderate morning preference (*p* = 0.046). Higher concentration of ATO induced higher *Bmal1* expression levels (*F* = 5.160, *p* = 0.028) in HDF cultures from ADHD participants with moderate evening chronotype, particularly at ZT12 after synchronization (Bonferroni post hoc test, *p* = 0.041) compared to the participants with definite evening type.

When comparing the two study groups, significant different *Clock* (*F* = 4.145, *p* = 0.010)*, Bmal1* (*F* = 4.815, *p* = 0.005) *and Per3* (*F* = 4.042, *p* = 0.011) expressions were observed in HDFs exposed to ATO collected from ADHD participants exhibiting neutral and moderate evening preference, as well as from healthy participants with morning preference. ADHD participants with moderate evening chronotype presented significant lower *Clock* (0.20 µM ATO, ZT16, Bonferroni post hoc test, *p* = 0.007) and *Bmal1* (0.58 µM ATO, ZT20, Bonferroni post hoc test, *p* = 0.015) expression levels compared to HC with definitive morning and moderate morning chronotype, respectively. ADHD participants with neutral type revealed higher *Per3* expression (0.58 µM ATO, ZT28, Bonferroni post hoc test, *p* = 0.011) than HC with moderate morning chronotype.

## Discussion

The results of the present study illustrate that attention-deficit hyperactivity disorder (ADHD) are associated to alterations in the circadian rhythm. It demonstrates that atomoxetine (ATO) impacts on circadian function, particularly the *Clock*, *Bmal1* and *Per2* gene expression.

*Ex vivo* expression of circadian genes in fibroblasts has previously been demonstrated to be a useful approach to study circadian rhythms related to sleep/wake behavior (Brown 2005; Hida 2017) in psychiatric disorders and particularly ADHD (Coogan et al. 2019; Johansson et al. 2011, 2016). ADHD is frequently linked with sleep disorders such as obstructive sleep apnea, peripheral limb movement disorder, restless legs syndrome and circadian-rhythm sleep disorders (Hvolby 2015).

Previously, we reported that patients with ADHD using ADHD-medication, such as methylphenidate and ATO, have altered sleep activity compared to both controls and ADHD participants without medication (Coogan et al. 2019). In the current study, measures from the non-parametric circadian rhythm analysis between both groups, healthy controls and ADHD participant showed no statistical significant effect of group for mid-sleep on weekend days, mid-sleep on weekdays, social jetlag, sleep WASO and total number of wake bouts.

ADHD individuals without medication showed alterations in the expression of *Per2* and *Cry1* compared to medicated ADHD patients or HC. *Clock* expression was altered in patients with ADHD using methylphenidate and ATO, but showed no significant rhythmicity (Coogan et al. 2019).

The incubation of HDF cultures collected from ADHD participants to 0.58 µM ATO, induced the rhythmicity of *Clock,* whereas this effect was not observed in other HDF cultures. It is to mention that previously several studies reported that *Clock* features minimal circadian variation. No significant 24 h variation in *Clock* expression was observed in oral mucosa and skin biopsies obtained from healthy participants. *Clock* was found to be arrhythmic in mice liver (Bjarnason 2001; Bonaconsa et al. 2014). Hansen et al. (2016) investigated if rhythmicity of clock- and metabolic gene expression is altered in human primary myotubes. The study groups were represented by type 2 diabetic patients with BMI- and age-matched obese controls, and lean, healthy, young endurance trained athletes with their age-matched sedentary controls. Hansen et al. (2016) confirmed no circadian rhythmicity for *Clock* gene expression whereas *Bmal1, Cry1, Per2* and *Per3* showed significant circadian rhythmicity in the study groups (Hansen et al. 2016).

In the present study, the observed ATO effect in the ADHD group on *Clock* rhythmicity could suggest a potential link between the norepinephrine and circadian rhythm pathways. ATO, as a selective NET inhibitor, modulates norepinephrine. Several studies have shown that norepinephrine is a synchronizer of the circadian rhythm (Chalmers et al. 2008; Li and Cassone 2015; Maletic et al. 2017; Morioka et al. 2010). Moreover, 48 h norepinephrine synchronization of pineal gland cultures from rats, induced a rhythmic expression pattern of the clock genes (Andrade-Silva et al. 2014). Preliminary results from our studies show that *Clock* gene rhythmicity is linked to higher body mass index (BMI). In the present study, there were no significant differences in BMI values across the two study groups.

Cultures from HC exposed to 0.20 µM ATO had a higher *Per1* and *Per3* expression, 12 h after dexamethasone synchronization. Paradoxically, the expression of the two period genes was lower in cultures exposed to higher ATO concentration. This observations suggests that lower dose of ATO is inducing a phase delay of the *Per1* and *Per3* gene expression, as well as for *Cry1*. However, this effect was not observed in the cultures derived from ADHD participants. The expression and rhythm of all genes except *Cry1* in the ADHD group adjusted to the healthy control group after ATO incubation. This effect is observed particularly for the lower ATO concentration. Higher concentration of ATO adjusts the expression of *Clock* gene toward the HC group, and shifts the expression of *Bmal1* and alters the expression of *Per3.*

We observed changes in the rhythmic expression of *Bmal1* and *Per2* genes. In ADHD patients and HC, *Bmal1* expression differed between the two ATO concentrations 16 and 28 h after dexamethasone synchronization. Moreover, 0.20 µM ATO induced different *Bmal1* expression between controls and ADHD group at ZT28. In HC, the expression of *Per2* at ZT12 was significant higher in HDF cultures incubated with 0.20 µM ATO compared to those exposed to 0.58 µM ATO. The expression of *Per2* was lower at ZT4 compared to negative control cultures from ADHD participants.

O’Keeffe et al. (2012) reported that ATO treatment at circadian time ZT6 induced a downregulation of CLOCK in the suprachiasmatic nucleus, but did not alter the expression of PER2 and BMAL1 (O’Keeffe et al. 2012).

Single nucleotide polymorphisms (SNP) in circadian genes were associated with core ADHD symptoms, increased evening-orientation and frequent sleep problem (Korman et al. 2018). Particularly *Per3* gene has been associated with chronotype, sleep homeostasis and various psychiatric disorders (Archer et al. 2018; Liberman et al. 2018). However, several studies report no association between the *Per3* VNTR and *Clock* SNP, diurnal preference and sleep/wake behavior (An^ 2014^; Hida^ 2018^; McGowan et al. 2020).

A limitation of the study is the small sample size. Further studies with a higher number of participants are necessary. It is to mention, that no special cognitive testing was implemented in this study. In addition, the participants of the ADHD group took no medication before and during the study. For further studies, a connection between circadian disturbances, cognitive deficits and the effect of medication would be suitable.

In the present study, strong positive correlations for chronotype and clock genes were observed for *Bmal1, Per3* and *Cry1* among the study participants, particularly after atomoxetine incubation at ZT0 and ZT16. Comparing the circadian preferences, statistical significant different *Clock, Bmal1 and Per3* expressions were observed in HDFs exposed to ATO in ADHD participants exhibiting neutral and moderate evening preference, as well in healthy participants with morning preferences. ADHD participants with evening preference have higher *Per2* expression 4 and 20 h after dexamethasone synchronization compared to the other chronotypes. We also observed an overexpression for *Clock* 16 h after synchronization for ADHD participants with moderate evening compared to HC with definitive morning preference. In summary, our results suggest that ATO influences circadian gene expression.

## Data Availability

Data and material are available.

## Code availability

Actiwatch 2, Philips Respironics, USA; CircWave v. 1.4 software (generated by Dr. Roelof Hut; www.euclock.org); SPSS (IBM Corporation).
